# Mucolytic treatment of chronic rhinosinusitis in a murine model of primary ciliary dyskinesia

**DOI:** 10.3389/fmolb.2023.1221796

**Published:** 2023-07-24

**Authors:** Weining Yin, Hannah L. Golliher, Amy J. Ferguson, Julia S. Kimbell, Alessandra Livraghi-Butrico, Troy D. Rogers, Barbara R. Grubb, Adam J. Kimple, Lawrence E. Ostrowski

**Affiliations:** ^1^ Marsico Lung Institute, University of North Carolina at Chapel Hill, Chapel Hill, NC, United States; ^2^ Department of Otolaryngology—Head and Neck Surgery, University of North Carolina at Chapel Hill, Chapel Hill, NC, United States; ^3^ Department of Pediatrics, University of North Carolina at Chapel Hill, Chapel Hill, NC, United States

**Keywords:** chronic rhinosinusitis, mucus, primary ciliary dyskinesia, nasal, computed tomography, sinus, CT, PCD

## Abstract

**Background:** Genetic defects in motile cilia cause primary ciliary dyskinesia (PCD), a rare disease with no specific therapeutics. Individuals with PCD often have impaired fertility and laterality defects and universally suffer from upper and lower airway diseases. Chronic rhinosinusitis is a universal feature of PCD, and mucus accumulation and subsequent infections of the sinonasal cavity cause significant morbidity in individuals with PCD. Despite this, there are no approved treatments that specifically target mucus.

**Objective:** The goals of this study were to determine whether computed tomography (CT) imaging could be used to quantify mucus accumulation and whether the use of a mucolytic agent to reduce disulfide cross-links present in mucins would improve the effectiveness of nasal lavage at removing mucus in a murine model of PCD.

**Methods:** Adult mice with a deletion of the axonemal dynein Dnaic1 were imaged using CT scanning to characterize mucus accumulation. The animals were then treated by nasal lavage with saline, with/without the disulfide-reducing agent tris(2-carboxyethyl)phosphine. Post-treatment CT scans were used to quantify improvement in the sinonasal cavity.

**Results:** Mucus accumulation in the nasal cavity was readily quantified by CT. Compared to sham-treated control animals, nasal lavage with/without a mucolytic agent resulted in a significant reduction of accumulated mucus (*p* < 0.01). Treatment with the mucolytic agent showed a greater reduction of accumulated mucus than treatment with saline alone.

**Conclusion:** The results suggest that inclusion of a mucolytic agent may increase the effectiveness of nasal lavage at reducing mucus burden in PCD.

## Introduction

Primary ciliary dyskinesia (PCD) is a rare genetic disease, usually inherited in an autosomal recessive manner, that occurs with an estimated incidence of 1 in ∼7,500 individuals (Hannah et al., 2022). The disease primarily affects the upper and lower respiratory tracts, where the lack of efficient mucociliary clearance (MCC) results in recurrent and chronic infections. Bronchiectasis, otitis media, and chronic rhinosinusitis (CRS) are common features of the disease (Knowles et al., 2016). Although advancements in sequencing technology have continued to identify the genetic causes of PCD, with over 50 different causal genes now confirmed, there has been little progress on the development of specific treatments to alleviate or eliminate the symptoms of the disease (Zariwala et al., 2015).

CRS is a common and debilitating disease symptom among individuals with PCD (Davis et al., 2015; Shapiro et al., 2016). Due to the lack of effective MCC, mucus accumulates in the sinuses and nasal passages, resulting in a significant decrease in the quality of life (Lucas et al., 2015) (Dell et al., 2016). In addition, mucus serves as a nidus for infection and may act as a reservoir of pathogens that lead to lower airway infections. Surprisingly, there are no currently approved mucolytic agents that have been demonstrated to effectively clear excess mucus from the nasal cavity. The only approved mucolytic agent, N-acetylcysteine (NAC), thins mucus by reducing disulfide bonds which polymerize mucin macromolecules (Pedre et al., 2021). However, the activity and absorption profile of NAC make it largely ineffective on the airway epithelium. Additionally, NAC has a noxious “rotten egg” odor that induces bronchospasms (Dano, 1971; Crouch et al., 2007). As an alternative to NAC, investigators have recently explored the effectiveness of tris(2-carboxyethyl)phosphine (TCEP) in models of muco-obstructive airway disease (Ehre et al., 2018; Morgan et al., 2021). We hypothesize that novel mucolytics may have therapeutic benefits in CRS. To test this hypothesis, we first explored the use of CT imaging to quantify the extent of mucus accumulation in an inducible mouse model of PCD that develops severe CRS (Ostrowski et al., 2010). We then performed a pilot study to examine the ability of nasal lavage with TCEP to improve mucus clearance from the nasal cavity.

## Methods

### Animal model

Animals were group-housed with a 12-h light/dark cycle and given free access to food and water. Experimental animals were generated as previously described (Ostrowski et al., 2010). Briefly, animals (3–4 weeks old) that were homozygous for the floxed allele of *Dnaic1* and heterozygous for Rosa/CreER (Dnaic1flox/flox/CreER^+/−^) were treated with tamoxifen (five intraperitoneal injections of 75 μg/g body weight; one injection given every 2–3 days) to induce a deletion in *Dnaic1*. The animals were aged for a minimum of six additional weeks after tamoxifen treatment to allow for the cessation of MCC and the development of rhinosinusitis and were usually studied at 4–6 months of age (Ostrowski et al., 2010). The animals were euthanized by CO_2_ asphyxiation. All studies used littermates of both sexes and were performed under protocols approved by the Institutional Animal Care and Use Committee of the University of North Carolina.

### Nasal lavage

For mucolytic or saline treatment, the animals (*n* = 29 each) were lightly anesthetized with isoflurane (drop method) and treated by nasal lavage with 20 μL of buffered saline with or without the addition of 10 mM TCEP. Small drops of the solutions (∼5 μL) were placed on the nares with an adjustable pipette and inhaled spontaneously. The animals were treated three times with a 90 min recovery time between treatments. Control animals (*n* = 14) were treated with anesthesia only (sham).

### CT scanning

CT images were obtained by the Biomedical Research Imaging Center at the University of North Carolina. Briefly, the animals were anesthetized with 2% isoflurane and scanned using a GE eXplore CT 120. The volume of the nasal airspace was quantified using image analysis software (Mimics, Materialise, Plymouth, MI). CT images were imported into Mimics, and contrast was set to bone scale. Thresholding (−1024 to 0) was used to create a mask including the airspace in the nasal cavity. The image was manually cropped at the tip of the nasal cavity and the beginning of the nasopharynx to isolate the nasal cavity. Additionally, isolated air-filled spaces were manually added to the airspace in the main nasal cavity. The Mimics software generated a 3D model, and the volume of the nasal airspace was measured. Volumes before treatment were subtracted from the volumes after treatment to determine the increase in airspace, representing the removal of mucus.

### Histology

Mouse heads were fixed in 10% buffered formalin and decalcified, and paraffin sections were prepared at three different levels of the nasal cavity, as previously described (Ostrowski et al., 2010). The sections were stained with hematoxylin and eosin or alcian blue–periodic acid–Schiff (to visualize mucus accumulation).

### Statistics

Statistical analysis was preformed using Prism 9.0 (GraphPad, San Diego, CA). A paired *t*-test was used to compare nasal airspace between animals pre- and post-treatment, and a one-way ANOVA with Tukey’s multiple comparisons was used to compare treatment groups. A mixed-model two-way ANOVA was utilized to compare repeated measures over time.

## Results

**FIGURE 1:**
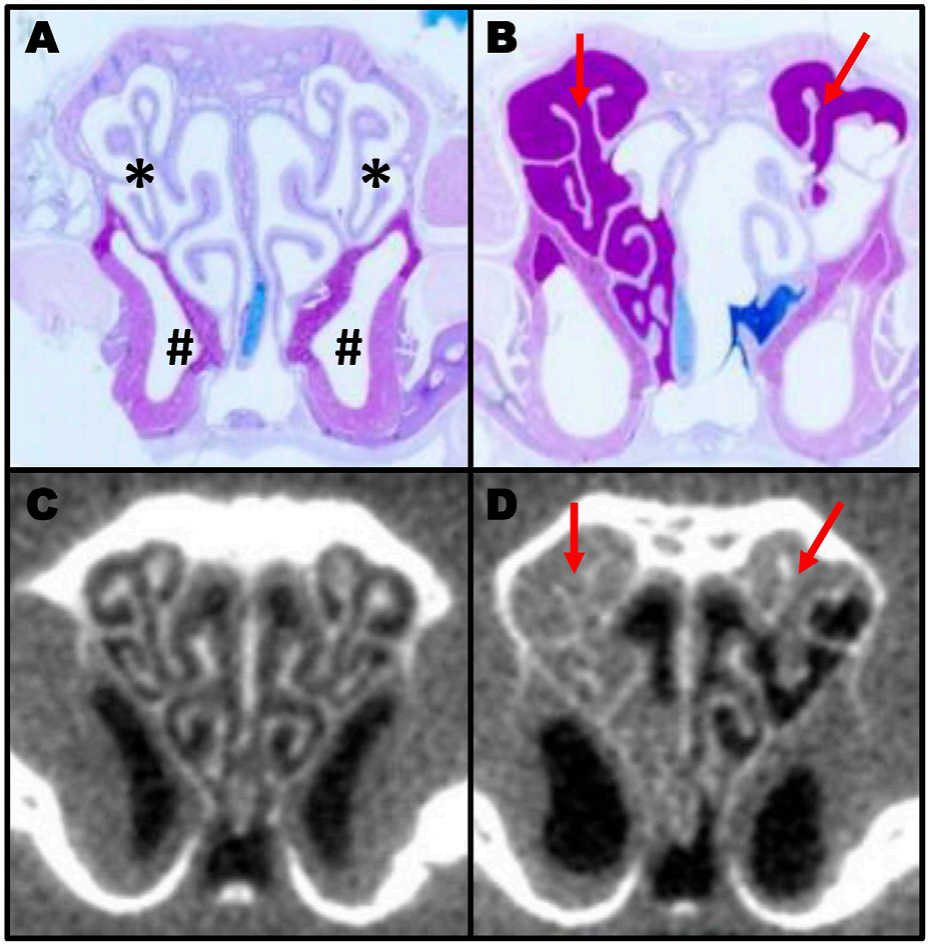
Comparison of histology section and CT scans in murine sinusitis. AB-PAS-stained section of a control **(A)** and a PCD **(B)** mouse showing mucus accumulation (red arrows) in the PCD animal. The same animals were imaged by CT **(C,D)** prior to euthanasia to illustrate the correlation between CT scanning and histology for measuring mucus obstruction (A; * indicates the ethmoturbinate region; # indicates the maxillary sinus region).

**FIGURE 2:**
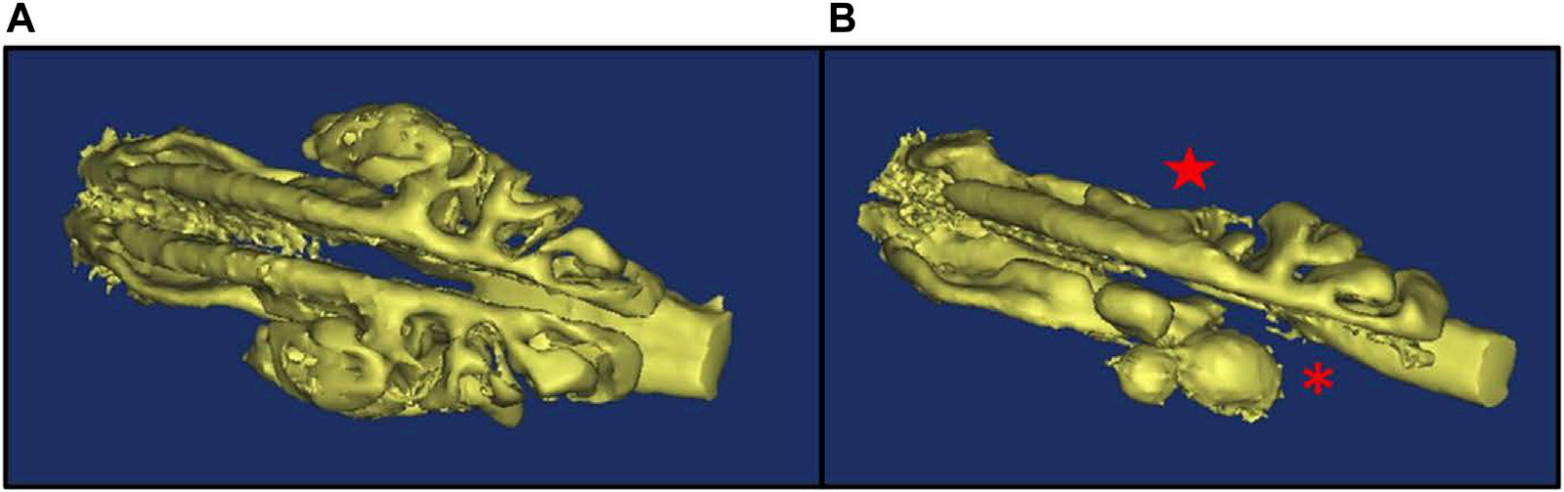
3D model of nasal airspace showing mucus accumulation in PCD mice. mCT imaging was used to obtain a 3D model of the nasal airspace in a control **(A)** and a PCD **(B)** mouse. Mucus accumulation in the PCD mouse is evidenced by the absence of airspace in the ethmoturbinate (asterisk) and maxillary sinus (star) regions.

To study the pathogenesis and treatment of PCD, we previously developed an inducible mouse model that avoids hydrocephalus and heart defects that occur in traditional knock-out models (Supplementary Figure S1) (Lee and Ostrowski, 2021) (Li et al., 2015). As previously reported, deletion of the ciliary protein Dnaic1 in a mouse model of PCD results in a loss of MCC and the subsequent accumulation of mucus and neutrophils in the sinonasal cavity (Ostrowski et al., 2010). However, postmortem histologic examination of the nasal cavity is limited to a single time point per animal. To evaluate the utility of CT scans to assess murine mucosal inflammation and mucus accumulations, we obtained CT scans of the nasal cavity from control and PCD mice and then prepared routine histological sections from the same animals. Examination of multiple sections from multiple animals demonstrated that the AB-PAS staining of mucus accumulation was visually concordant with the murine CT scans (Figure 1). Furthermore, CT scanning allowed the construction of a 3D model and quantification of the airspace of the entire nasal cavity (Figure 2), compared to the 2D limitation of traditional histology sections.

**FIGURE 3:**
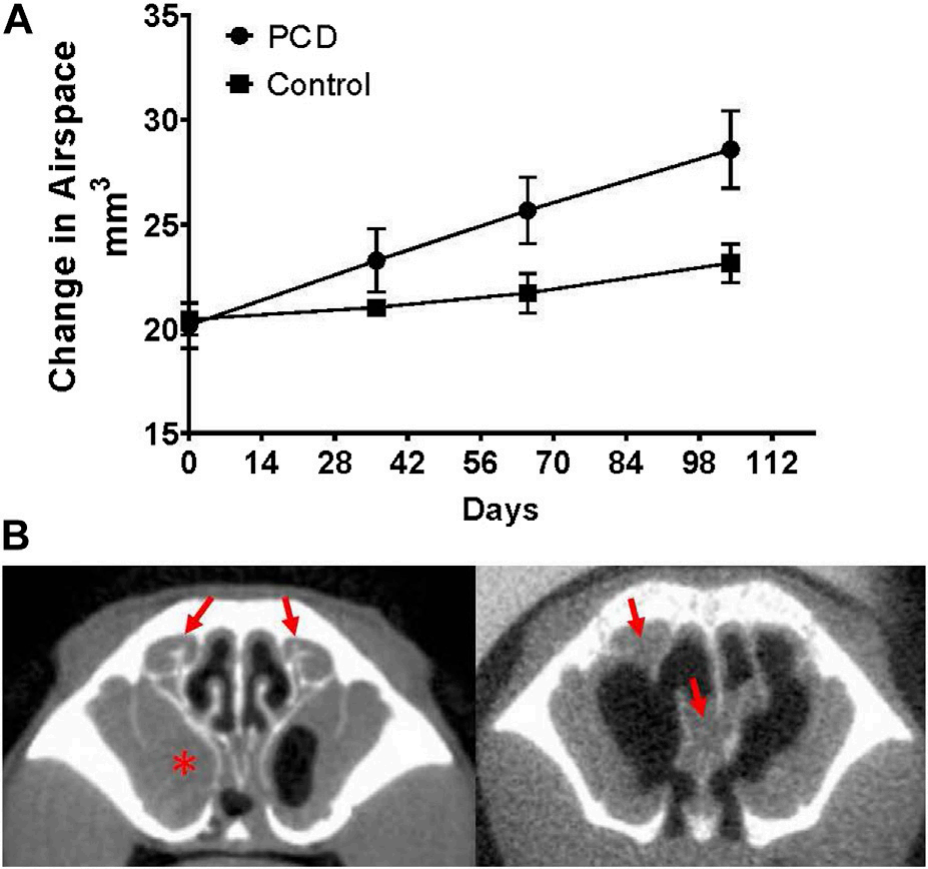
Changes in nasal airspace measured by CT imaging over time. **(A)** Groups of PCD (*n* = four males and three females) and littermate control mice (*n* = two males and two females) were repeatedly imaged by CT at monthly intervals beginning at ∼7 weeks of age. Although PCD mice demonstrate mucus accumulation in the nasal cavity over this time, the nasal airspace increases compared to the control (*p* = 0.0415). **(B)** Representative images of a mouse with mucus accumulation (left; arrows indicate mucus accumulation in the ethmoturbinate region, and asterisk indicates the mucus-occluded sinus cavity) compared to a mouse with severe remodeling/degeneration resulting in an increase in nasal airspace, even though accumulated mucus is still present (arrows).

We therefore used CT imaging to further examine the pathogenesis of CRS in this model. A group of PCD animals was imaged by CT over several months, and the change in nasal airspace was monitored by constructing 3D models. Although the PCD animals routinely showed evidence of accumulated mucus, the airspace of the sinonasal cavity increased over time in the PCD animals compared to the controls (*p* = 0.0415; Figure 3A). This expansion was due to degeneration of the turbinates and enlargement of the sinuses (Figure 3B).

To begin investigating the effectiveness of reducing agents for the treatment of CRS, several pilot studies were performed. PCD mice were imaged by CT to obtain a baseline measure of nasal airspace and then treated by intranasal lavage with 10 mM TCEP. Several treatment protocols were tested (e.g., 1–3 doses daily; 1–5 days). Following treatment, a repeat CT scan was obtained, and the change in nasal airspace was measured. Some of the treated animals showed large areas of mucus clearing, as indicated by the increase in nasal airspace (Supplementary Figure S2).

**FIGURE 4:**
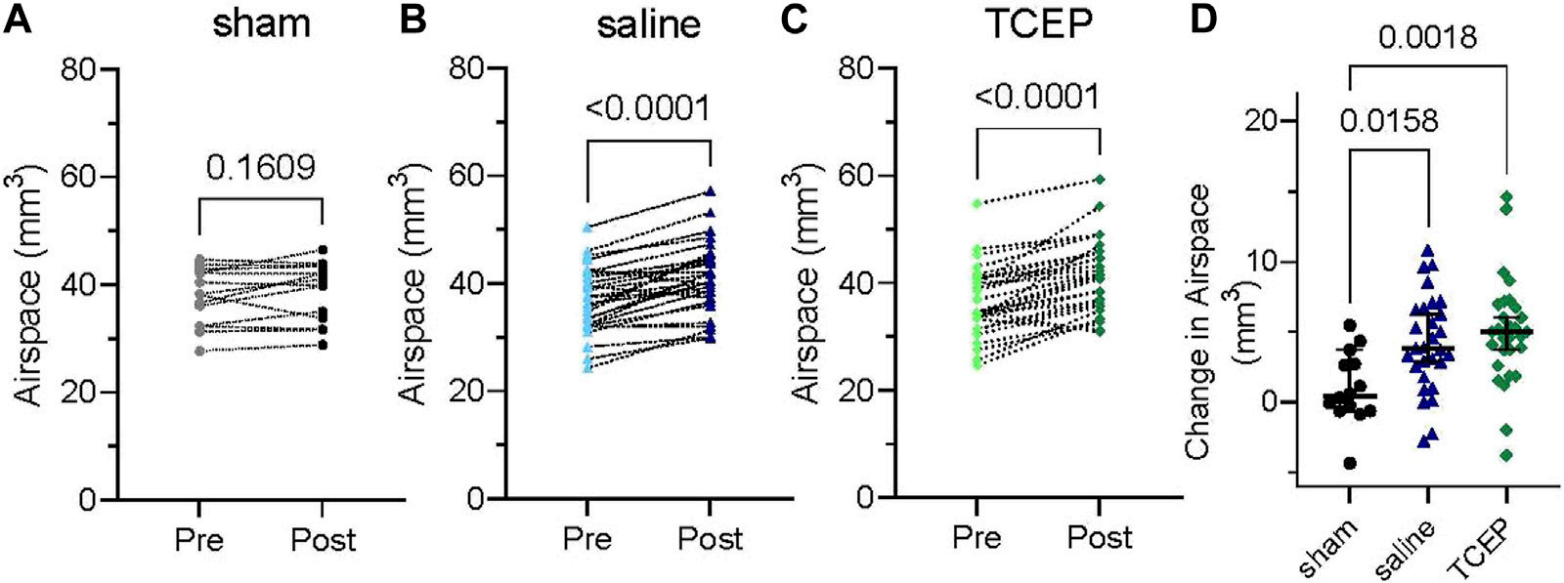
Saline and TCEP lavage improve nasal airspace in a murine model of PCD compared to sham treatment. Groups of PCD mice were treated three times by nasal lavage, and the change in nasal airspace was measured. **(A)** At baseline, mice in the sham group had a mean airspace of 37.8 mm^3^ (95% C.I. 34.7–40.8 mm^3^) and 38.8 mm^3^ after sham treatment (95% C.I. 35.6–42.0 mm^3^) with no significant difference (*p* = 0.1609). **(B)** Saline treatment increased nasal airspace from 37.0 mm^3^ (95% C.I. 34.6–39.3 mm^3^) to 41.1 mm^3^ (95% C.I. 38.6–43.7 mm^3^; *p* < 0.0001). **(C)** Treatment with TCEP increased nasal airspace from 36.4 mm^3^ (95% C.I. 33.9–38.9 mm^3^) to 41.4 mm^3^ (95% C.I. 38.7–44.0 mm^3^; *p* < 0.0001). **(D)** TCEP treatment showed a greater increase in nasal airspace than saline or sham.

To further examine the effectiveness of reducing agents for the acute treatment of CRS, we used a protocol similar to what individuals might utilize in a home or clinical setting. Groups of PCD mice were imaged by CT to obtain a baseline measure of nasal airspace. The following day, the animals were treated three times by intranasal lavage with 10 mM TCEP or saline (20 μl; 90 min between treatments) and again imaged by CT. The change in nasal airspace was determined by an investigator blinded to the animals’ treatment. As an additional control, some animals (sham) underwent a mock treatment (anesthesia only). Not surprisingly, the sham-treated animals showed no significant change in nasal airspace (Figure 4A; *p* = 0.1609; *n* = 14). In contrast, both TCEP- and saline-treated animals showed a highly significant increase in nasal airspace (Figures 4B, C; *p* < 0.0001; *n* = 29). Compared to sham, saline-treated animals showed an improvement in nasal airspace of 3.2 mm^3^ (*p* = 0.0158), while TCEP-treated animals showed a larger 4.0 mm^3^ improvement of the nasal airspace over sham treatment that was highly significant (*p* = 0.0018) (Figure 4D). TCEP-treated animals showed an overall improvement of 0.81 mm^3^ compared to saline treatment (*p* = 0.64).

## Discussion

CRS causes substantial morbidity, impairs quality of life, and may seed the lower respiratory tract in individuals with PCD (Lucas et al., 2015; Dell et al., 2016). In this genetic cause of CRS, mucus accumulation is the inciting factor for disease pathogenesis, and the development of therapies to reduce or remove mucus would likely improve patient outcomes. At present, the only approved mucolytic agent, N-acetylcysteine (NAC), has not been proven effective at improving mucus clearance. Thus, the testing and further development of improved mucolytic agents is needed.

To study the pathogenesis and treatment of PCD, we have previously developed an inducible murine model of PCD. This model avoids situs abnormalities and hydrocephalus that frequently occur in traditional knock-out models of PCD, including the knock-out model of *Dnaic1* (Supplementary Figure S1), and allows for the study of adult animals (Ostrowski et al., 2010). Inducing the deletion of the ciliary protein Dnaic1 in post-natal mice results in a loss of MCC and mucus accumulation in the nasal cavity. Thus, this animal model may be useful for the testing of mucolytic agents.

Herein, we present data demonstrating that the accumulation of mucus and the subsequent remodeling of the nasal cavity can be visualized and quantified by CT scans (Figure 1). Not surprisingly, comparing the images obtained by CT with the routine histological staining of nasal sections demonstrated good visual concordance, indicating that quantifying the volume of nasal airspace could be used as an inverse measure of mucus accumulation. Interestingly, we observed an increase in nasal airspace over time as a consequence of disease progression, due to remodeling of the nasal cavity (Figure 3). The mechanisms responsible for this remodeling are unknown and will require further investigation.

We then explored the use of this model to test the effect of a mucolytic agent on mucus clearance. Nasal lavage with saline is commonly used by individuals suffering from CRS due to PCD or other causes. Nasal lavage is proposed to clear mucus and debris and provide symptomatic relief. In these studies, saline alone reduced the mucus burden and significantly increased nasal airspace compared to sham-treated controls (*p* = 0.0084). These results confirm the beneficial effects of lavage and provide clear evidence that the murine model of PCD will be useful to investigate mucolytic treatments of CRS. Inclusion of a mucolytic agent (TCEP) in the lavage resulted in a greater improvement in nasal airspace compared to animals treated with saline alone (5.0 vs. 4.2 mm^3^). Although not statistically significant, treatment with the mucolytic agent showed a clear trend toward more efficient mucus removal (Figure 3D), potentially by reducing the disulfide cross-links in the mucin molecules. Similarly, Ehre et al. (2018) demonstrated a decrease in lung mucus burden in a mouse model of obstructive lung disease, and Morgan et al. (2021) demonstrated a decrease in mucus burden in an allergic mouse model following treatment with a reducing agent. Taken together, these studies suggest that mucolytic agents may be useful in the treatment of CRS caused by PCD or other diseases.

In summary, our studies demonstrate the usefulness of the murine model and CT imaging for studies of CRS in PCD. Furthermore, our results suggest that additional studies of mucolytic agents, exploring different treatment regimens and/or improved mucolytic agents, are warranted.

## Data Availability

The raw data supporting the conclusion of this article will be made available by the authors, without undue reservation.

## References

[B1] Crouch B. I. Caravati E. M. Dandoy C. (2007). Effect of dilution with beverages on the smell and taste of oral acetylcysteine. Am. J. Health Syst. Pharm. 64 (18), 1965–1968. 10.2146/ajhp060568 17823110

[B2] Dano G. (1971). Bronchospasm caused by acetylcysteine in children with bronchial asthma. Acta Allergol. 26 (3), 181–190. 10.1111/j.1398-9995.1971.tb01294.x 5109293

[B3] Davis S. D. Ferkol T. W. Rosenfeld M. Lee H. S. Dell S. D. Sagel S. D. (2015). Clinical features of childhood primary ciliary dyskinesia by genotype and ultrastructural phenotype. Am. J. Respir. Crit. Care Med. 191 (3), 316–324. 10.1164/rccm.201409-1672OC 25493340 PMC4351577

[B4] Dell S. D. Leigh M. W. Lucas J. S. Ferkol T. W. Knowles M. R. Alpern A. (2016). Primary ciliary dyskinesia: First health-related quality-of-life measures for pediatric patients. Ann. Am. Thorac. Soc. 13 (10), 1726–1735. 10.1513/AnnalsATS.201603-198OC 27464304 PMC5122491

[B5] Ehre C. Rushton Z. L. Wang B. Hothem L. N. Morrison C. B. Fontana N. C. (2018). An improved inhaled mucolytic to treat airway muco-obstructive diseases. Am. J. Respir. Crit. Care Med. 199, 171–180. 10.1164/rccm.201802-0245OC PMC6353008 30212240

[B6] Hannah W. B. Seifert B. A. Truty R. Zariwala M. A. Ameel K. Zhao Y. (2022). The global prevalence and ethnic heterogeneity of primary ciliary dyskinesia gene variants: A genetic database analysis. Lancet Respir. Med. 10 (5), 459–468. 10.1016/S2213-2600(21)00453-7 35051411 PMC9064931

[B7] Knowles M. R. Zariwala M. Leigh M. (2016). Primary ciliary dyskinesia. Clin. chest Med. 37 (3), 449–461. 10.1016/j.ccm.2016.04.008 27514592 PMC4988337

[B8] Lee L. Ostrowski L. E. (2021). Motile cilia genetics and cell biology: Big results from little mice. Cell Mol. Life Sci. 78 (3), 769–797. 10.1007/s00018-020-03633-5 32915243 PMC7902362

[B9] Li Y. Klena N. T. Gabriel G. C. Liu X. Kim A. J. Lemke K. (2015). Global genetic analysis in mice unveils central role for cilia in congenital heart disease. Nature 521 (7553), 520–524. 10.1038/nature14269 25807483 PMC4617540

[B10] Lucas J. S. Behan L. Dunn Galvin A. Alpern A. Morris A. M. Carroll M. P. (2015). A quality-of-life measure for adults with primary ciliary dyskinesia: QOL-PCD. Eur. Respir. J. 46 (2), 375–383. 10.1183/09031936.00216214 25976687 PMC4522020

[B11] Morgan L. E. Jaramillo A. M. Shenoy S. K. Raclawska D. Emezienna N. A. Richardson V. L. (2021). Disulfide disruption reverses mucus dysfunction in allergic airway disease. Nat. Commun. 12 (1), 249. 10.1038/s41467-020-20499-0 33431872 PMC7801631

[B12] Ostrowski L. E. Yin W. Rogers T. D. Busalacchi K. B. Chua M. O'Neal W. K. (2010). Conditional deletion of dnaic1 in a murine model of primary ciliary dyskinesia causes chronic rhinosinusitis. Am. J. Respir. Cell Mol. Biol. 43 (1), 55–63. 10.1165/rcmb.2009-0118OC 19675306 PMC2911571

[B13] Pedre B. Barayeu U. Ezeriņa D. Dick T. P. (2021). The mechanism of action of N-acetylcysteine (NAC): The emerging role of H(2)S and sulfane sulfur species. Pharmacol. Ther. 228, 107916. 10.1016/j.pharmthera.2021.107916 34171332

[B14] Shapiro A. J. Zariwala M. A. Ferkol T. Davis S. D. Sagel S. D. Dell S. D. (2016). Diagnosis, monitoring, and treatment of primary ciliary dyskinesia: PCD foundation consensus recommendations based on state of the art review. Pediatr. Pulmonol. 51 (2), 115–132. 10.1002/ppul.23304 26418604 PMC4912005

[B15] Zariwala M. A. Knowles M. R. Leigh M. W. (2015). in Primary ciliary dyskinesia. Editor Pagon R. A. (Seattle (WA): GeneReviews).

