# Supplementary figures and images for: Mucolytic treatment of chronic rhinosinusitis in a murine model of primary ciliary dyskinesia
Source: Front Mol Biosci. 2023 Jul 24;10:1221796. doi: 10.3389/fmolb.2023.1221796 (PMC10405821; doi:10.3389/fmolb.2023.1221796)

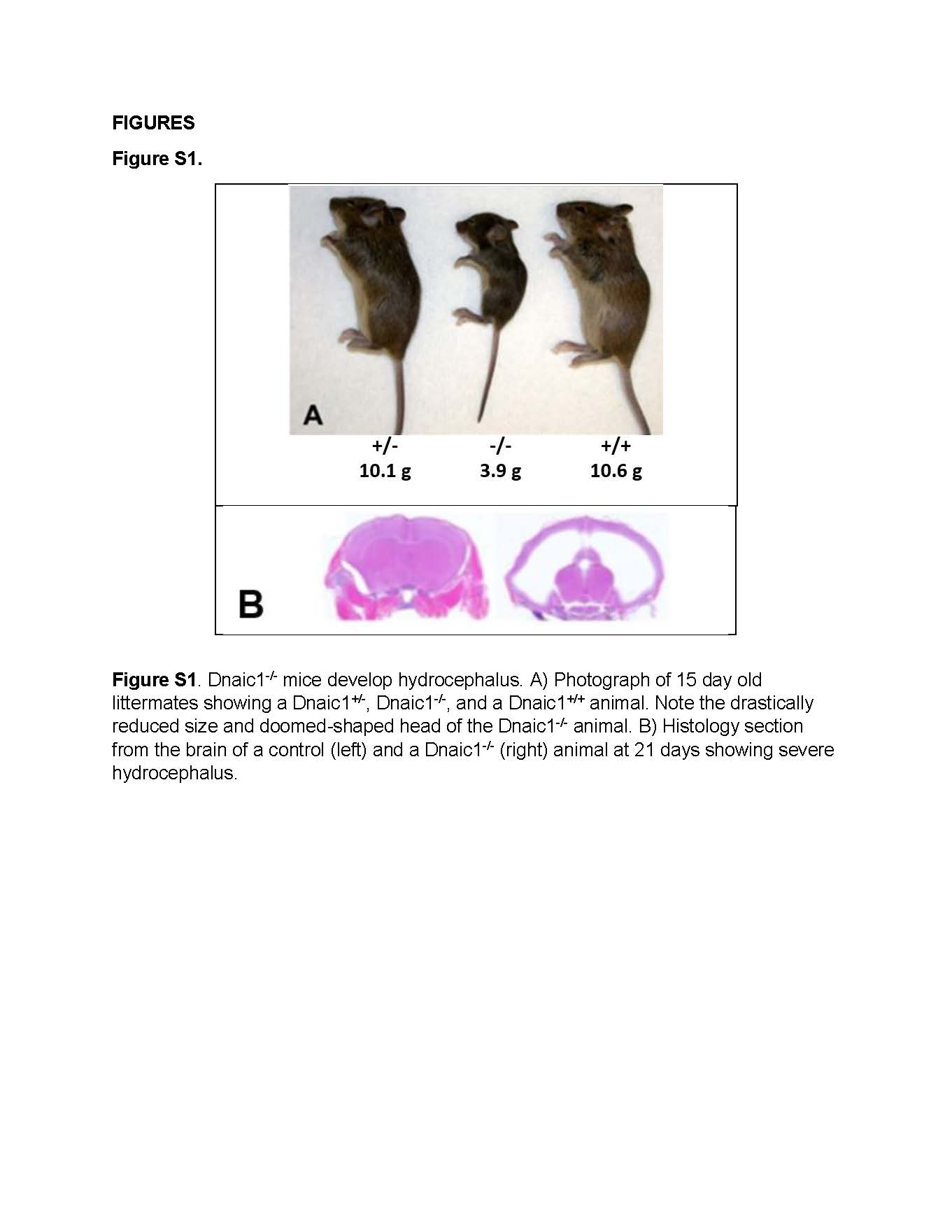

Supplement: Supplementary file 1 [file Image1.JPEG]

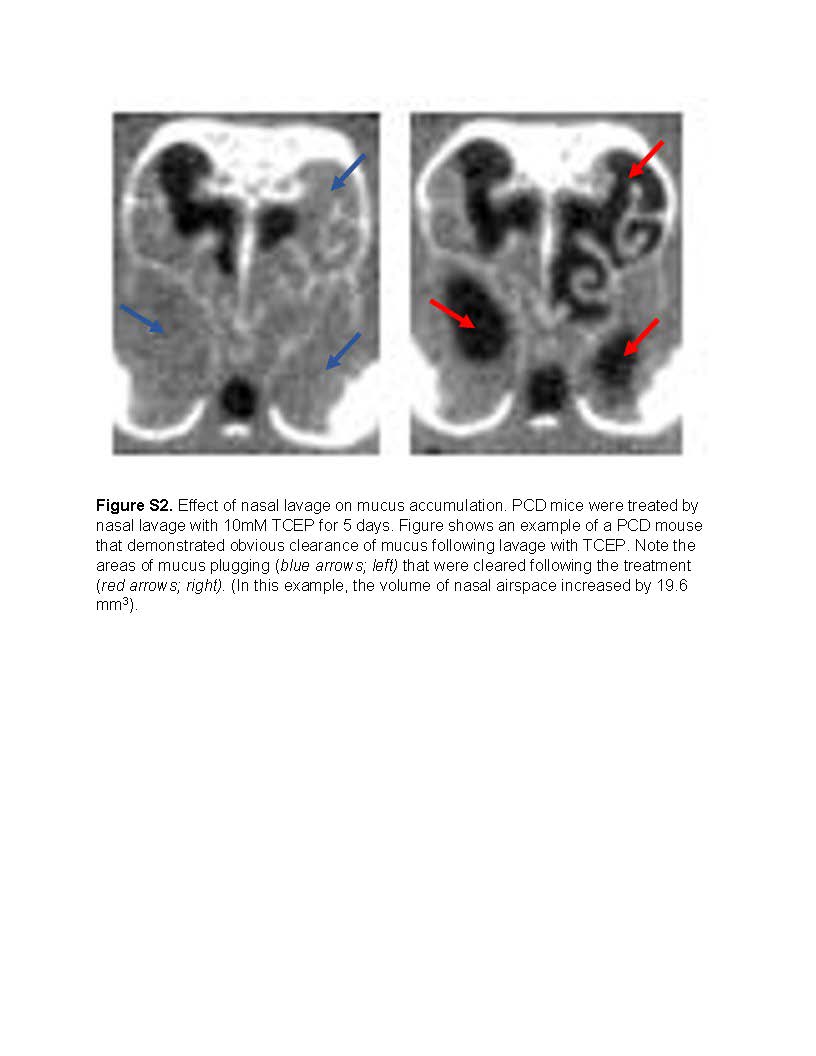

Supplement: Supplementary file 2 [file Image2.JPEG]
